# United States-based practice guidelines for children and adolescents with eating disorders: Synthesis of clinical practice guidelines

**DOI:** 10.1186/s40337-025-01254-6

**Published:** 2025-04-11

**Authors:** Cara Bohon, Daniel Le Grange, Evelyn Attia, Neville H. Golden, Dori Steinberg

**Affiliations:** 1 Equip Health, Carlsbad, CA USA; 2 Department of Psychiatry and Behavioral Sciences, Stanford University, Stanford, CA USA; 3 Department of Psychiatry and Behavioral Sciences, University of California, San Francisco, San Francisco, CA USA; 4 Department of Psychiatry and Behavioral Neuroscience (Emeritus), The University of Chicago, Chicago, IL USA; 5 Department of Psychiatry, Columbia University Medical Center, New York, NY USA; 6 Department of Psychiatry, Weill Cornell Medical College, New York, NY USA; 7 Department of Pediatrics, Division of Adolescent Medicine, Stanford University, Stanford, CA USA

**Keywords:** Eating disorders, Practice guidelines, Anorexia nervosa, Bulimia nervosa, Binge eating disorder, Avoidant/restrictive food intake disorder

## Abstract

**Introduction:**

Several practice guidelines exist from professional organizations in the United States to support the assessment and management of eating disorders in children and adolescents. This manuscript synthesizes the key areas of overlap from these guidelines and provides directions for future research and alignment to improve care.

**Recommendations:**

Consistent screening for eating disorders in primary care is recommended to ensure early identification and referral to treatment. Outpatient treatment supported by families, including family based treatment, is the first line of care recommended by guidelines. Multidisciplinary treatment teams benefit patients in covering the variety of aspects of health that eating disorders impact, including mental health, nutritional health, and physical health. Patients may require hospitalization under certain medical criteria such as bradycardia or specific lab abnormalities.

**Conclusions:**

Guidelines show consensus on the importance of early identification and treatment access, involvement of family in treatment, and the use of a multidisciplinary treatment team. However, future work is needed to guide care of Avoidant/Restrictive Food Intake Disorder (ARFID), as well as the impact of weight inclusive care and the development of validated screening tools for children and adolescents for all eating disorders.

## Abstract (plain language summary)

Several professional organizations in the U.S. provide guidelines for assessing and treating eating disorders in children and adolescents. This paper highlights key areas of agreement across these guidelines and suggests future research directions.

## Key Recommendations

Primary care providers should routinely screen for eating disorders to enable early detection and treatment referrals.

Outpatient care, particularly family-based treatment, is the preferred first-line approach.

Multidisciplinary treatment teams that include therapists, dieticians, and medical professionals are essential for comprehensive care.

Hospitalization may be necessary for patients with severe medical complications.

## Introduction

Thirty million Americans will have an eating disorder (ED) during their lifetime [1]. EDs have high morbidity and mortality rates [2], and onset generally occurs during adolescence [3]. Early identification and referral to evidence-based treatments leads to improved outcomes [4–6].

## Methods of guideline development

Several professional organizations in the United States (US) developed guidelines that address the identification and management of EDs in children and adolescents. These are the American Psychiatric Association (APA) [7], the Society for Adolescent Health and Medicine (SAHM) [8], the American Academy of Pediatrics (AAP) [9], and the American Academy for Child and Adolescent Psychiatry (AACAP) [10]. In June 2023, The Kennedy Forum and Equip Health brought together leaders from these organizations and other stakeholders, such as health systems, payors, policy advocates, and individuals with lived experience with an ED for a summit to review current guidelines and align on opportunities for future areas of research. Below we synthesize the guidelines from these organizations as presented at this summit, supplemented with recommendations for future research that arose during the summit, summarized by the authors. We first summarize the recommendations within the published guidelines and then provide the subsequent recommendations thereafter.

### Guideline recommendations

#### Screening of EDs should occur regularly

All practice guidelines address screening and referral for treatment for children and adolescents. AAP recommends that pediatricians screen for EDs during all annual health visits [9], and SAHM emphasizes the importance of early identification and management without specificity for timing of screening [8]. APA and AACAP recommend screening for the presence of an eating disorder as part of every initial psychiatric evaluation regardless of age or presenting problem [10]. While the US Preventive Services Task Force found insufficient evidence to support routine screening for EDs in asymptomatic adolescents and young adults, there is general consensus that medical providers should suspect the diagnosis of an ED in the presence of significant weight deviations (using growth curve data), presence of disordered eating behaviors such as restricting, purging, binge eating, or food avoidance, presence of amenorrhea, exercise-related injuries (e.g., stress fractures), lab abnormalities (e.g., hypokalemia), or low resting heart rate (HR < 50) [7, 8, 10, 11].

#### >Outpatient treatment should be the first-line treatment for most patients

Guidelines recommend patients should be treated in the least restrictive setting that can appropriately address medical and co-occurring needs [7, 8, 10, 11]. For most patients, outpatient treatment is the appropriate first-line treatment; however, for some patients, higher levels of care (partial hospital programs, residential treatment, hospital admission, etc.) may be needed as an initial treatment. All guidelines are aligned for when higher levels of care are needed and provide criteria for when hospital-based treatment may be indicated for medical complications and/or severe co-occurring psychiatric conditions [7, 8, 10, 11]. Pharmacological interventions should be used primarily for comorbid conditions, as existing randomized controlled trials of psychotropic medications for eating disorders have been conducted in adult samples [7, 8, 10, 11].

#### Treatment is aimed at medical stabilization and normalization of eating behaviors

In some patients with vital sign instability, electrolyte disturbances, ECG abnormalities or severe malnutrition, hospitalization for acute medical stabilization is needed and guidelines from the different organizations are in general agreement regarding factors justifying medical hospitalization. Treatment next prioritizes normalization of eating behaviors as the primary means of restoring physical and mental health. The specific treatment approach will depend on the patient’s diagnosis. However, some patients will require weight restoration and nutritional rehabilitation including increases in the quantity, frequency and/or variety of dietary intake. As a best-practice for relapse prevention, most patients require ongoing ED-informed treatment following several months of sustained weight recovery and/or cessation of ED behaviors [7].

#### Family-based treatment (FBT) has the strongest evidence base in children and adolescents

All guidelines state evidence-based treatments should be employed and specifically recommend FBT for youth with anorexia nervosa (AN) or bulimia nervosa (BN). Emerging evidence suggests that FBT may also be helpful in treating avoidant/restrictive food intake disorder (ARFID) and binge eating disorder (BED) [12–14]. Cognitive behavioral therapy (CBT) and dialectical behavioral therapy (DBT) are described as potential adjunctive modalities for older adolescents and young adults [15].

#### Target (or treatment goal) weight range should be individualized

ED treatment goals for those who are at a low weight often include weight restoration, for which providers must set a goal weight range for recovery. Most guidelines acknowledge that EDs occur across the weight spectrum and cite the limitations of using the readily measurable absolute body mass index (BMI) to determine target weight, especially in children and adolescents [7, 8, 10, 11]. APA guidelines in particular discuss how BMI does not measure body composition or account for racial and sex differences [7]. For children and adolescents, BMI percentile from growth charts should be used and target weight range determination should be guided by individual growth charts, stage of pubertal development and growth trajectory, and could be higher or lower than median BMI.

#### Comorbidities should be treated concurrently

Eating disorders frequently co-occur with anxiety disorders, mood disorders, obsessive-compulsive and related disorders, trauma and stress related disorders, and substance-use disorders [9]. Symptoms of depression and anxiety may ameliorate after full nutritional rehabilitation and sustained weight recovery [7, 16]. However, as co-occurring disorders can trigger or exacerbate ED symptoms, all practice guidelines recommend treating comorbidities utilizing evidence-based treatments [17].

#### Multidisciplinary care teams are recommended

EDs are multi-system illnesses requiring multidisciplinary care, as recommended across all guidelines. Team members may include a mental health provider, a medical provider, psychiatrist, and a registered dietitian [8].

**Table 1:** Points for the assessment and management of eating disorders as stated by the Society for Adolescent Health and Medicine (SAHM) in 2022, American Psychiatric Association (APA) in 2023, the American Academy of Pediatrics (AAP) in 2021, and the American Academy for Child and Adolescent Psychiatry (AACAP) in 2015

	SAHM	APA	AAP	AACAP
Outpatient is first line unless clinically indicated	x	x		x
Specific lab and diagnostic tests recommended		x	x	x
Clinical assessments are necessary to determine level of care	x	x		
Provides specific factors supporting hospitalization	x	x	x	x
Treatment plan should be determined		x		
Provides pharmacological guidance		x	x	x
Outcomes need to be measured to determine effectiveness		x		
Multidisciplinary care teams	x	x	x	x
Coordination of care	x	x	x	
Weekly weight gain goals and/or overall target weight goals for those that need weight restoration	x	x		
Employ evidence-based treatments (e.g., FBT for adolescents)	x	x	x	x
Family involvement for adolescents with AN and BN	x	x	x	x
Screening for the presence of an eating disorder as part of an initial evaluation	x	x	x	x

SAHM = Society for Adolescent Health and Medicine, APA = American Psychiatric Association, AAP = American Academy of Pediatrics, AACAP = American Academy for Child and Adolescent Psychiatry, FBT = family-based treatment, AN = anorexia nervosa, BN = bulimia nervosa

### Summary and considerations for future research

The synthesis of US-based ED practice guidelines, indicates much consensus across organizations in the treatment of EDs for children and adolescents. However, practice guidelines are guided by currently available research, and we outline several areas below that warrant further study.

#### Standards for screening

The importance of screening in primary care settings was specified across guidelines; however, no validated screening tools exist for children and adolescents. Further, the US Services Preventive Task Force evaluated the ‘balance of benefits and harms’ for adult and adolescent screening across conditions, and evidence was deemed insufficient to make a determination for eating disorders [18]. Thus, the evidence statement is not a recommendation for or against screening, but rather that there is insufficient evidence to make a recommendation [19]. Moreover, primary care providers are often the first point of contact, yet physician training to recognize an ED is limited at best, particularly in non-stereotypical presentations of EDs [20]. Future research on best screening tools for children and adolescents is vital to ensure primary care providers have optimal tools and recommendations for best practice. Additionally, research on the best approach for implementation of screening into clinical practices will be valuable.

#### **Ensure care is culturally relevant for marginalized groups**,** non-stigmatizing**,** and considers social determinants of health**

Individuals from marginalized groups such as individuals who identify as Black, Indigenous, and people of color or LGBTQ+, and cisgender boys and men have EDs at equal or greater rates compared with white, cisgender girls, yet they receive care at lower rates and are more likely to leave treatment early [1]. Both diagnostic and treatment studies need more diverse representation [21], so that guidelines can make inclusive statements on treatments. Factors related to stigma or social determinants of health also place patients at risk of care disparities. Issues include patient reluctance to report symptoms, failure of patients and providers to recognize symptoms as an ED, limitations of public insurance, underrepresentation of diverse providers, financial and geographic barriers, and lack of access to trained specialists. Future guidelines should include the impact of these issues on ED identification and treatment.

#### Address weight stigma and ensure weight-inclusive care

The prototypical image of a patient with an ED is someone presenting with anorexia nervosa who is at a very low weight. Consequently, although EDs occur across the weight spectrum, patients at higher weights are less likely to receive timely diagnosis and treatment. Similarly, illness severity is more accurately reflected by the relative amount, duration, and rapidity of weight loss rather than by a low body weight [22–24]. Clinicians should assess the degree of recent weight loss because medical complications (e.g., refeeding syndrome) are predicted by both the rapidity and total magnitude of weight loss [22, 25]. Additional studies are needed to inform recommendations for level of care and medical interventions for individuals with severe illness at higher weights.

#### ARFID warrants specific treatment guidelines

The diagnosis avoidant/restrictive food intake disorder (ARFID) was added to the Diagnostic Statistical Manual-Fifth Edition (DSM-5) list of Feeding and Eating Disorders in 2013^3^. The prevalence of ARFID, which typically has its onset in childhood [26], is similar to other well-known diagnoses (AN, BN), and current long-term outcomes for malnourished patients with ARFID are similar to childhood-onset AN [27]. Patients often present with longstanding malnutrition, with one-third requiring hospitalization [28]. Most guidelines offer minimal direction for ARFID as there were few or no treatment trials at the time the guidelines were written. Studies are now underway that will allow guidelines to include more detailed information for medical providers on differentiating between ARFID symptoms versus ‘age-appropriate’ picky eating behaviors. Treatment of comorbid psychiatric issues also needs to be addressed, as the majority of patients with ARFID present with at least one comorbidity [26]. Research is also needed on advances in pharmacological, nutrition, and therapeutic modalities for ARFID.

In sum, while there is much consensus across existing US practice guidelines prioritizing early outpatient, evidence-based treatment, such as FBT, and using individualized target weights as treatment goals, there are still important gaps to be addressed in future versions.

## Data Availability

No datasets were generated or analysed during the current study.
